# Valorization of Grape Pomace: A Review of Phenolic Composition, Bioactivity, and Therapeutic Potential

**DOI:** 10.3390/antiox13091131

**Published:** 2024-09-19

**Authors:** Anna Karastergiou, Anne-Laure Gancel, Michael Jourdes, Pierre-Louis Teissedre

**Affiliations:** Univ. Bordeaux, INRAE, Bordeaux INP, Bordeaux Sciences Agro, UMR 1366, OENO, ISVV, F-33882 Villenave d’Ornon, France; anna.karastergiou.1@u-bordeaux.fr (A.K.); anne-laure.gancel@u-bordeaux.fr (A.-L.G.); michael.jourdes@u-bordeaux.fr (M.J.)

**Keywords:** grape pomace, polyphenols, valorization, health benefits, antioxidant activity, anti-inflammatory properties, metabolism

## Abstract

*Vitis vinifera* L., commonly known as grapes, is one of the most widely cultivated crops worldwide, with over 80% used for wine production. However, the winemaking process generates substantial residues, including grape pomace (GP), wine lees, and wastewater, which can pose significant environmental and economic challenges. Among these, GP stands out not only as a waste product but also as a rich source of polyphenols—bioactive compounds with recognized antioxidant and anti-inflammatory properties. Recent advancements have expanded the application of GP-derived extracts, particularly in the health and food industries, due to their potent bioactive properties. This review provides a comprehensive overview of the valorization of GP, focusing on its phenolic composition and therapeutic potential. It evokes innovative, environmentally friendly extraction techniques and integrated methods for the chemical analysis of these valuable compounds. Additionally, the health benefits of GP polyphenols are explored, with recent experimental findings examining their metabolism and highlighting the key role of gut microbiota in these processes. These insights contribute to a deeper understanding of the biological activity of GP extracts and underscore their growing significance as a high-added-value product. By illustrating how winemaking by-products can be transformed into natural therapeutic agents, this review emphasizes the importance of sustainable development and eco-friendly waste management practices, significantly contributing to the advancement of a circular economy.

## 1. Introduction

Grapes (*Vitis vinifera* L.) are among the world’s most significant fruit crops, with nearly 7.2 million hectares of vines cultivated globally. In 2023, the production of wine from grapes reached approximately 258 million hectoliters, reflecting the dynamic and vital aspect of the global wine industry [1]. Italy, France, and Spain are the leading wine-producing countries, contributing to nearly half of the world’s total wine production [2,3]. In 2023, Italy produced approximately 47.5 million hectoliters, followed by France with 42.2 million hectoliters, and Spain with 35.7 million hectoliters.

However, the winemaking process generates important amounts of by-products. Grape pomace (GP), the main viticultural residue, accounts for about 20–30% of the total grape mass [4]. For example, for every 1000 kg of processed grapes, around 750 L of wine and 130 kg of GP are produced [5], resulting in an annual waste of approximately 9 million tons [6].

GP consists of skins (~50%), seeds (~25%), and stalks (~25%) [7]. Its typical composition includes cellulose, sugars, fats, organic acids, polyphenols, proteins, fibers, pectins, and other compounds such as minerals and vitamins in smaller proportions (Table 1).

However, the discard of GP can pose significant environmental and economic challenges [8]. Improper disposal can lead to soil and groundwater contamination and severe health risks to aquatic and human populations. Consequently, the management of GP is a critical concern for the wine industry [6].

Recent research has focused on the valorization of GP as a sustainable resource, emphasizing the extraction of valuable compounds like polyphenols, which remain abundant even after vinification. Only 30–40% of phytochemicals transfer from grapes to wine during winemaking, leaving the rest in the pomace [9,10]. Most of these phenolic compounds are found in the seeds (about 60–70%), 30–35% in the skins, and the remaining in the pulp [11].

Therefore, GP can present both a challenge and an opportunity. Many studies have reported that GP can be efficiently utilized in various sectors, including animal feed and biofuel production, and as a source of valuable polyphenols for food, nutraceutical, and cosmetic products. These applications not only provide economic value but also offer significant environmental benefits by reducing waste and promoting sustainable practices in the wine industry [12,13].

By exploiting the rich bioactive compounds in GP, industries can develop new, high-value products while addressing the environmental issues associated with pomace disposal. This aligns with the broader goals of sustainable development and circular economy, making GP a valuable resource for future innovations [14].

The objective of this review is to provide a thorough examination of the valorization of GP phenolic compounds, with a focus on recent advancements in their extraction, chemical analysis, and characterization. While many studies have examined the phenolic content of GP, this review stands out by highlighting the latest innovations in green extraction technologies that enhance yield and efficiency, alongside precise chemical analysis methods for profiling these bioactive compounds.

Furthermore, a crucial aim of this work is to emphasize the bioactivity and health benefits of GP polyphenols, particularly their potential as therapeutic agents. The antioxidant and anti-inflammatory properties of GP extracts across various biological systems are thoroughly examined and novel insights into the metabolism of GP polyphenols are presented, addressing a research area that remains relatively unexplored. Recent findings on how gut microbiota influence the metabolic pathways of these compounds are discussed, highlighting their role in enhancing bioavailability and therapeutic efficacy. This represents a significant, though preliminary, advancement in understanding the mechanisms behind GP’s health-promoting effects, offering new perspectives on its potential in treating and preventing various health conditions.

Overall, this review aims to make a meaningful contribution to the scientific discourse on the valorization of by-products, providing new insights and identifying future research directions, particularly in the application of GP as a valuable source of health-promoting compounds.

**Table 1:** Grape pomace composition [13,14,15,16,17].

Component	Amount
**Moisture (water)**	50–60%
**Dietary fiber**	20–30%
**Carbohydrates**	5–15%
**Protein**	8–15%
**Polyphenols**	5–10%
**Lipids**	3–7%
**Organic acids**	3–6%
**Ash**	3–5%

## 2. Review Conceptualization

In this study, several databases covering biomedical and pharmaceutical sciences were analyzed, including Pubmed, Scopus, SciFinder, Scholar, and ISI Web of Knowledge. The databases were accessed in June 2024. A literature search was conducted for the period from January 2010 to June 2024. The query strings combined the following terms: “grape pomace”, “grape marc”, “grape byproduct”, “winemaking waste”, “phenolic compounds”, “polyphenols”, “valorization”, “health benefits”, “antioxidant activity”, “anti-inflammatory properties”, and “metabolism”, and the final selection of relevant studies was made by crossing combinations of these terms.

A total of 4846 documents were initially retrieved from the databases for the specified time range. After applying inclusion and exclusion criteria based on relevance to the research objectives, 586 documents were selected for further review. For the final selection, priority was given to recent, high-impact studies, particularly those from the past five years. The review focused on research that closely aligned with its primary objective—providing a detailed examination of GP polyphenols, with a particular focus on their chemical characterization, biological activities, and metabolic processes. These aspects form the core of the investigation and are critical to advancing the understanding of the bioactive potential of GP.

## 3. Grape Pomace Phenolic Composition

*Vitis vinifera* grapes are particularly rich in polyphenols and their by-product, GP, retains a high content of these compounds, as previously mentioned. Polyphenols are a diverse group of secondary metabolites found abundantly in various plant sources, including fruits, vegetables, herbs, and seeds, making them the most prevalent phytochemicals in the plant kingdom [18]. These compounds are characterized by a common structure that includes an aromatic ring with one or more phenol units [19]. The synthesis of polyphenols occurs through the phenylpropanoid pathway, a crucial biosynthetic route in plants that converts phenylalanine into a variety of polyphenolic compounds via enzymatic reactions involving key intermediates such as cinnamic acid and p-coumaric acid [20].

To date, more than 10,000 phenolic compounds have been identified, broadly categorized into two major groups: flavonoids and non-flavonoids [21]. This wide chemical diversity arises from the various forms these compounds can take—free or conjugated—differing by their hydroxylation level and by modifications such as glycosylation, methylation, or acylation [22].

Polyphenols play multiple roles in plant physiology. They contribute to plant defense against abiotic stresses (ultraviolet (UV) radiation, salinity, drought, heavy metals, etc.), as well as biotic stresses from herbivores, bacteria, and fungi, and aid in plant nutrition and reproduction [23]. Their protective effects are largely due to their potent antioxidant properties [24]. Consequently, these compounds have gained considerable attention in recent decades for their health benefits, with studies focusing on various in vitro and in vivo systems, including human cell lines, animal models, and microbial cultures, to evaluate their antibacterial, anti-inflammatory, anti-cardiovascular, anti-diabetic, and anti-cancer activities [25]. Moreover, several clinical trials have been conducted to validate their therapeutic potential, particularly in the context of anti-cancer and anti-diabetic effects, demonstrating promising results in improving patient outcomes [26,27,28,29].

Grape varieties are often categorized based on their skin color, which plays a significant role in determining the phenolic profile. Grapes can generally be classified into red, white, and black/purple varieties. Red grapes, such as *Cabernet Sauvignon* and *Merlot*, are rich in anthocyanins, which contribute to their deep color and have strong antioxidant properties [30,31]. They also contain high levels of flavonols and tannins, known for their anti-inflammatory and cardioprotective effects. In contrast, white grapes, such as *Chardonnay* and *Sauvignon Blanc*, have lower anthocyanin and tannin content but are still rich in phenolic acids, like caffeic acid and p-coumaric acid, which contribute to their antioxidant capacity [32]. Black or purple grapes, like *Concord*, are abundant in resveratrol, a stilbene compound well known for its potential anti-aging and anti-cancer properties [33,34].

The phenolic composition of GP can vary significantly depending on factors such as soil type, agro-climatic conditions, grape variety, and winemaking techniques [17,35]. More specifically, the winemaking process differs for red and white wines. In white wine production, grapes are crushed, and the must is usually not fermented with the solid residues. In contrast, red wine production involves maceration and alcoholic fermentation of grape berries with the must [8]. This difference significantly affects the composition of the pomace, with white varieties, although less rich in phenolics, still offering valuable unfermented by-products.

**Figure 1:**
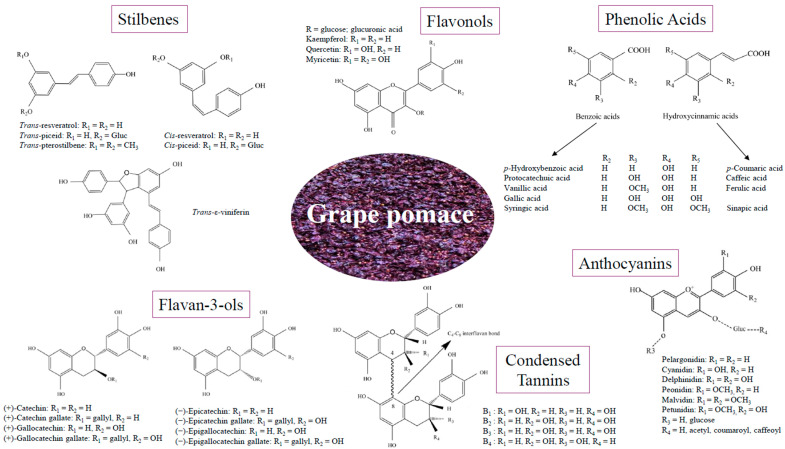
Main phenolic compounds found in grape pomace.

Diverse phenolic families can be found in GP, including flavonoids, phenolic acids, and stilbenes [36,37,38] (Figure 1).

### 3.1. Flavonoids

**Figure 2:**
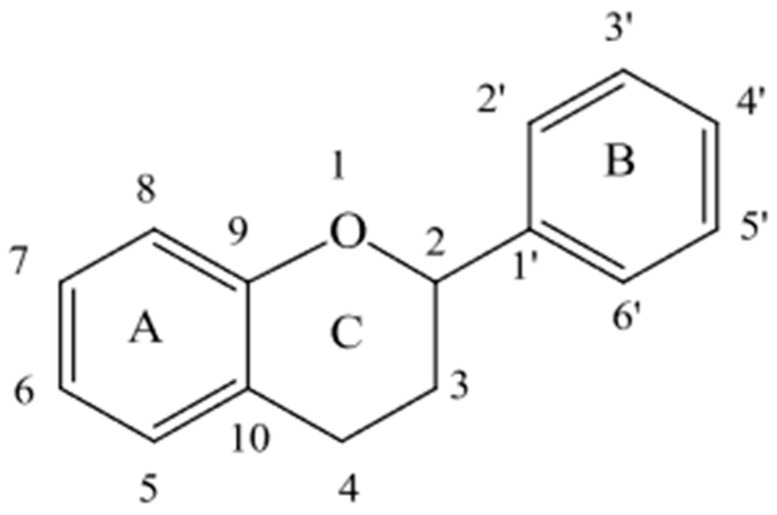
Flavonoids’ basic structure. The carbon atoms are numbered on rings A (positions 5–9), B (positions 1’-6’), and C (positions 2–4, with 10 at the junction of A and C). The oxygen atom is located at position 1 in ring C.

Flavonoids are a significant group of polyphenols defined by their C6-C3-C6 structure, which consists of two phenyl rings (A and B) connected by a three-carbon bridge that forms a closed heterocyclic pyran ring (C) (Figure 2). This phenyl benzopyran skeleton allows for a wide range of substitutions and modifications, resulting in various flavonoid families [39,40]. The primary flavonoids found in GP are flavanols, anthocyanins, and flavonols [17]. Some studies also identify the presence of other flavonoid groups, such as flavanones and flavones, in GP. However, due to their low concentrations, the references in the literature are very limited [8,41].

#### 3.1.1. Flavan-3-ols

Flavan-3-ols are flavonoid compounds that play a crucial role in the nutritional and sensory properties of grapes and grape-derived products. Chemically, flavanols are differentiated from other flavonoids by a hydroxyl group (–OH) attached to the third carbon atom of the C ring. These compounds are present in both grape seeds and skins, with seeds typically containing a higher concentration and a different composition [42]. Flavan-3-ol monomers include catechin and epicatechin, which can further form oligomers and polymers such as condensed tannins or proanthocyanidins. Additionally, flavanols can be esterified with gallic acid, forming compounds like catechin gallate, epicatechin gallate, gallocatechin, epigallocatechin, and epigallocatechin gallate [43].

Flavan-3-ols are among the most common phytochemicals found in GP. Numerous studies have reported significant levels of monomers like catechin and epicatechin, as well as several procyanidins, the main group of condensed tannins composed of these flavanol monomeric units [44,45,46,47]. Compounds such as epigallocatechin, and, less commonly, gallocatechin, catechin gallate, and epicatechin gallate, have also been characterized in GP [48,49,50].

A recent study investigated the phenolic composition of *Vitis vinifera* Carménère pomace extracts obtained through hot water pressurized extraction (HWPE). The results indicated that higher extraction temperatures and the use of glycerol significantly enhanced the recovery of monomers, including catechin (203.60 ± 0.06 μg/g dry weight (DW)) and epicatechin (162.45 ± 0.03 μg/g DW), as well as epicatechin gallate (54.61 ± 0.06 μg/g DW). Procyanidin dimers B1 and B2, trimers C1 and B2 gallate, and procyanidin tetramer B2 di-gallate concentrations varied depending on the extraction conditions, with ranges between 3.64 ± 0.07 μg/g DW and 87.94 ± 0.04 μg/g DW [51].

Fontana et al. (2017) [12] conducted a comprehensive analysis of the phenolic profiles of pomace extracts from various Argentinian grape varieties, emphasizing the flavan-3-ol content. The study reported high levels of catechin and epicatechin, and significant amounts of procyanidin dimers B1 and B2, as well as gallocatechin, gallocatechin gallate, and epicatechin gallate, with concentrations varying depending on the grape variety.

#### 3.1.2. Anthocyanins

Anthocyanins are natural pigments responsible for the red, blue, and purple colors in many fruits and vegetables [52]. In grapes, these pigments are located exclusively in the skins. Anthocyanins primarily exist as glycosides, formed when an anthocyanidin, the aglycone (sugar-free) form, binds to one or more sugar molecules. This glycosylation enhances their stability and water solubility, making anthocyanins the main pigments responsible for vivid coloration in plant tissues.

Anthocyanidins, the structural core of anthocyanins, are based on the flavylium ion or 2-phenylbenzopyrylium and contain hydroxyl and methoxyl groups in various positions [11]. Numerous anthocyanidins are found in red grapes, varying according to the number and position of the hydroxyl and methoxyl substituents [53,54], with malvidin glucosides being the most abundant ones.

While the majority of anthocyanins are extracted into wine during vinification, a significant number remains in red GP. Anthocyanins identified in GP mainly include 3-O-glucosides of delphinidin, cyanidin, petunidin, pelargonidin, peonidin, and malvidin. Additionally, acetyl, coumaryl, or caffeoyl derivatives—modified forms of anthocyanins where acetic, coumaric, or caffeic acid groups are attached to the anthocyanin molecule—have also been identified. The significant health benefits of these molecules have led to numerous studies focusing on their chemical characterization in GP [7,55,56,57].

An interesting study by Oliveira et al. (2015) [58] has highlighted the anthocyanin composition of GP. Advanced techniques like liquid chromatography and mass spectrometry (LC-DAD/MS) as well as matrix-assisted laser desorption/ionization time-of-flight (MALDI-ToF) were employed to analyze red GP extracts from Portuguese grape varieties such as Touriga Nacional, Touriga Franca, Tinta Roriz, Tinta Barroca, and Tinto Cão. The authors identified over 50 anthocyanin-based pigments, demonstrating the diversity and complexity of anthocyanins in GP. Key findings included the detection of various pyranoanthocyanins, such as A- and B-type vitisins and methylpyranoanthocyanins, as well as oligomeric malvidin-3-O-coumaroylglucoside-based anthocyanins. These findings underscore the rich anthocyanin content in GP, highlighting its potential as a valuable source of natural pigments for the food, cosmetic, and pharmaceutical industries.

#### 3.1.3. Flavonols

Flavonols are distinguished by a 3-hydroxyflavone backbone and are synthesized exclusively in the skins of grape berries. They are less abundant compared to flavan-3-ols and anthocyanins. In fruits, flavonols play a crucial role in enhancing color by stabilizing the colored forms of anthocyanin molecules and protecting from UV radiation [59]. The primary flavonols in grapes include kaempferol, quercetin, myricetin, and their methylated derivatives isorhamnetin, laricitrin, and syringetin. Kaempferol and quercetin are present in both white and red grape varieties, while myricetin and isorhamnetin are specific to red grapes [60].

In grapes, these compounds are typically found as glucosides, galactosides, rhamnosides, and glucuronides [61]. However, during the winemaking and aging processes, acid hydrolysis occurs, leading to the presence of both free aglycones and glycosylated forms in wines [60]. This hydrolysis might also account for the detection of aglycon forms in GP, as reported by several studies [62,63,64,65].

### 3.2. Non-Flavonoids

Non-flavonoids are structurally simpler than flavonoids. They are characterized by their basic phenolic structure, which lacks the three-ring configuration typical of flavonoids [66]. This family includes phenolic acids and stilbenes, both of which play significant roles in the antioxidant properties of grapes and grape-derived products.

#### 3.2.1. Phenolic Acids

Phenolic acids are a diverse class of compounds, divided into hydroxybenzoic acids and hydroxycinnamic acids based on the number and position of hydroxyl groups on the aromatic ring [67,68]. Hydroxycinnamic acids are the predominant phenolic compounds in grapes, characterized by a C6-C3 carbon skeleton with a double bond in the side chain. The primary hydroxycinnamic acids found in GP include caffeic, ferulic, and p-coumaric acids [38]. These compounds often conjugate with tartaric acid, forming caftaric, fertaric, and coutaric acids, respectively [69]. Significant amounts of these compounds are also present in GP skins. Hydroxybenzoic acids are derivatives of benzoic acid, featuring a C6-C1 carbon skeleton. The most common hydroxybenzoic acids in GP are vanillic, syringic, gallic, protocatechuic, and ellagic acids [70].

Phenolic acids are abundant in GP, making it a valuable source of bioactive compounds. Studies have shown that the total phenolic acid content in GP can vary significantly. For example, a study by Onache et al. (2022) [13] found that syringic acid levels ranged from 18.31 to 19.80 mg/100 g in white GP and from 22.00 to 94.30 mg/100 g in red GP, with Merlot GP showing the highest amounts. Similarly, gallic and ellagic acids were found in comparable amounts in both white and red GP, with concentrations ranging from 6.44 to 14.02 mg/100 g for gallic acid and from 0.28 to 3.63 mg/100 g for ellagic acid. The previous literature has reported comparable quantities of syringic and gallic acids in GP, with gallic acid levels between 2.52 and 36.04 mg/100 g and syringic acid levels from 46.9 to 173.1 mg/100 g in Cabernet Sauvignon, Merlot, and Malbec red GP [10,71,72].

#### 3.2.2. Stilbenes

**Figure 3:**
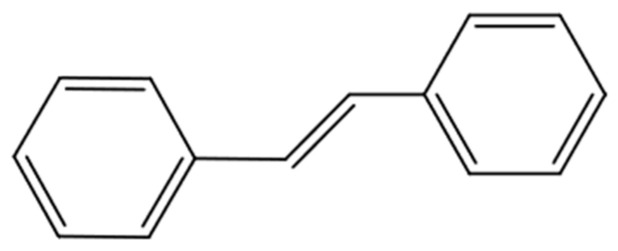
*Trans*-stilbene structure.

Stilbenes are phytoalexins, predominantly located in grape skins, with a chemical structure of C6-C2-C6, characterized by a 1,2-diphenylethylene backbone (Figure 3). Plants produce these compounds as a defense mechanism against biotic and abiotic stresses. Stilbenes can exist as monomers, dimers, or oligomers. Resveratrol is the most well-known stilbene, capable of forming more complex compounds by undergoing several modifications such as glycosylation, methoxylation, oligomerization, and isoprenylation [73]. The most abundant monomeric stilbenes in GP are trans-resveratrol and trans-piceid [35,46,74]. Caponio et al. (2022) [75], in their study, also reported the detection of ε-viniferin. Additionally, studies have reported the identification of ε-viniferin in other types of by-products such as vine canes and shoots [76,77].

## 4. Extraction Techniques for Polyphenols

**Figure 4:**
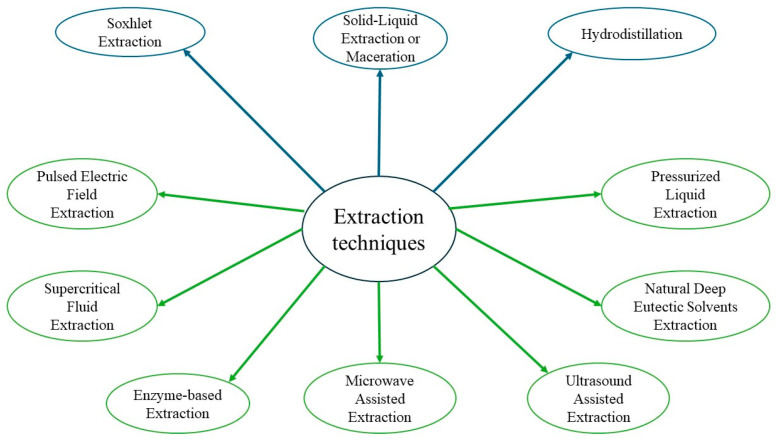
Classical (blue) and modern (green) extraction techniques.

Extraction is the primary method for obtaining phenolic compounds from raw materials like GP. The choice of the appropriate extraction technique is crucial, as it significantly impacts both the yield and recovery of these compounds. Classical extraction techniques with organic solvents have long been used and remain to date the most applied processes. However, modern, more innovative, sustainable, greener, and environmentally friendly alternatives are emerging for the optimal retrieval of polyphenols from GP (Figure 4).

### 4.1. Solid–Liquid Extraction

Solid–Liquid Extraction (SLE) is a conventional technique extensively used for extracting bioactive compounds from plant materials, including grapes and GP. Due to the polar nature of polyphenols, organic solvents such as ethanol, methanol, acetone, ethyl acetate, and chloroform are commonly employed. These solvents may not always selectively target the desired compounds, making double extractions with the same or different solvents common to enhance recovery rates [78,79].

The typical SLE process involves maceration and mechanical stirring. Key factors influencing SLE performance include solvent type, solvent-to-sample ratio, particle size, pH, extraction temperature, and extraction time [11]. However, optimizing these parameters can be time consuming due to the numerous combinations possible. Design of Experiments (DoE) appears to be a useful technique in this situation.

Response surface methodologies (RSM), empirical models based on mathematical and multivariate statistical techniques, allow for fewer trials and are often utilized for complex experimental processes that consider many factors.

Several studies have focused on the modeling and optimization of GP polyphenol extractions using RSM to conduct more efficient trials and increase extraction yields [7,80,81,82].

For instance, Rodrigues et al. (2023) [83] employed a Central Composite Design to optimize the extraction of phenolic compounds from GP using various solvents, including water, ethanol, acetone, methanol, and butanol. The study focused on four key factors: temperature, extraction time, liquid–solid ratio, and solvent concentration. The RSM model revealed that the quadratic effect of ethanol concentration was the most significant factor, followed by temperature. The optimal conditions were found to be 60 °C for ethanol extraction and 50 °C for acetone, 1.5 h, L/S ratio of 25 mL/g dry GP, and 50% ethanol concentration. The optimized conditions resulted in phenolic yields of 45.18 mg gallic acid equivalent (GAE)/g DW for acetone and 38.70 mg GAE/g DW for ethanol.

However, SLE techniques have significant drawbacks, including being time consuming and costly due to the high volume of solvents required, posing challenges regarding solvent disposal and recycling. Furthermore, ineffective extraction can occur due to compound loss through hydrolysis and oxidation [11].

### 4.2. Ultrasound-Assisted Extraction

Ultrasound-Assisted Extraction (UAE) uses low-frequency ultrasound waves (20 to 100 kHz) to induce cavitation within the plant matrix. Cavitation involves the formation and collapse of microbubbles, disrupting cellular walls and enhancing mass transfer across cell membranes. This improves solvent access to polyphenolic compounds and increases extraction efficiency [84,85]. Compared to conventional methods, UAE offers reduced processing time, lower solvent consumption, and the ability to extract heat-sensitive bioactive compounds at lower temperatures. Additionally, UAE is considered an eco-friendly and green technology, making it promising for industrial scale-up [86].

The effectiveness of UAE is influenced by factors such as ultrasound frequency and power, which can vary from 20 to 700 watts depending on the matrix and target molecules [11,87]. Publications highlight UAE’s efficiency in recovering compounds from vinification by-products. For example, González-Centeno et al. (2014) [88] optimized UAE conditions for Syrah GP using RSM. They studied the influence of acoustic frequency, ultrasonic power density, and extraction time on total phenolics, total flavonols, and antioxidant capacity. The optimal conditions achieved high yields of phenolic compounds and antioxidant activity, highlighting the effectiveness of UAE over traditional methods.

In a similar study, the influence of ultrasound power, citric acid concentration, and solid–liquid ratio was evaluated for the recovery of phenolic compounds from Syrah GP skins. The study found that UAE significantly enhanced the extraction efficiency, achieving a recovery of 59% of quantified phenolic compounds within just 3 min of processing, alongside an improvement in antioxidant capacity. The optimal conditions were determined to be 3000 W/L power, 2.5% citric acid, and a solid–liquid ratio of 1:15 [89].

González et al. (2020) [90] examined the UAE of bioactive compounds in fresh and freeze-dried Tannat GP. They assessed the influence of temperature, ultrasound power, and extraction time on the yield of total phenolic content (TPC), total monomeric anthocyanins, and total antioxidant capacity. The study revealed that UAE significantly enhanced extraction yields, with freeze-dried pomace showing a 180% increase in TPC compared to conventional methods.

### 4.3. Microwave-Assisted Extraction

Microwave-Assisted Extraction (MAE) operates by applying microwaves (0.3 to 300 GHz), creating localized heating that breaks down the plant matrix, and facilitates compound diffusion into the solvent. MAE enhances mass transfer from the interior to the exterior of the plant cell, improving extraction yields in a shorter period. Typically, microwave power ranges from 300 to 900 W and extraction temperatures from 50 to 100 °C. MAE reduces organic solvent use, has low costs, and is generally sustainable [91,92].

MAE’s challenge is maximizing extraction yield while avoiding damage to the chemical structure of natural compounds. It is effective for low-molecular-weight polyphenols like phenolic acids and flavonoids but less so for polymeric compounds like anthocyanins and condensed tannins due to the risk of degrading heat-sensitive compounds [93]. Several factors influence MAE efficiency, including microwave power and frequency, radiation duration, temperature, pressure, and the number of extraction cycles. The choice of solvent is crucial, as it affects both the solubility of target components and the absorption of microwave energy. RSM are commonly applied to optimize extraction conditions [62,94,95,96].

A recent application of microwave extraction for phenolic compounds is Microwave Hydrodiffusion and Gravity (MHG), a technique initially proposed for extracting essential oils [97]. This greener technique uses only water as the solvent. During hydrodiffusion, water molecules within the sample heat up due to dipolar rotation, causing localized heating that breaks plant cell walls. Despite its potential, excessive heating remains a limitation of MAE, particularly for thermolabile compounds [98,99].

Moro et al. (2021) [100] used MHG to extract phenolic compounds from GP, showing promising results with a max yield of 118.1 mg GAE/g of total phenolic compounds and significant antioxidant properties. Crescente et al. (2023) [101] compared UAE and MHG for GP from the Italian variety Aglianico, finding both methods effective. However, UAE produced higher total extracts, while MHG fractions exhibited higher antioxidant activities, especially in anthocyanin fractions tested on a human colorectal adenoma (HT-29) cell line.

### 4.4. Pressurized Liquid Extraction

Pressurized Liquid Extraction (PLE) is an advanced solid–liquid extraction technique that efficiently extracts bioactive compounds from plant materials. PLE methodologies include Accelerated Solvent Extraction (ASE), Enhanced Solvent Extraction, and Pressurized Hot Water Extraction [102].

PLE operates under high pressure (4 to 20 MPa), keeping the solvent liquid at elevated temperatures, enhancing solvent diffusion into plant pores and improving target compound release and solubility. The closed-system nature of PLE minimizes oxidation, making it suitable for extracting thermally sensitive phytochemicals. PLE is considered straightforward and versatile for phenolic extraction due to its compatibility with various solvents [103].

Temperature is crucial in PLE; increasing it from 40 °C to 120 °C improves phenolic extraction efficiency but can risk lower recovery rates of thermosensitive polyphenols [92,104]. PLE stands out as effective and sustainable for extracting polyphenols from GP, offering higher yields, reduced solvent volume, shorter extraction times, and selectivity compared to low-pressure methods. Its high level of automation makes it suitable for various industrial applications, such as the production of dietary supplements, functional food ingredients, and natural additives for cosmetics and pharmaceuticals [103].

Studies have shown the high effectiveness of PLE in recovering antioxidants and phenolic compounds, such as anthocyanins and proanthocyanidins, from GP [105,106]. In a study by Huamán-Castilla et al. (2020) [107], glycerol was introduced for the first time as a novel co-solvent for Pressurized Hot Water Extraction (PHWE). They evaluated various water–glycerol mixtures (15%, 32.5%, and 50%) using a PHWE system at temperatures of 90 °C, 120 °C, and 150 °C. The results showed that glycerol outperformed ethanol, yielding significantly higher amounts of polyphenols. This was attributed to glycerol’s ability to form additional hydrogen bonds with polyphenols.

### 4.5. Supercritical Fluid Extraction

Supercritical Fluid Extraction (SFE) utilizes the unique properties of fluids in a supercritical state—where temperature and pressure exceed critical values—to extract bioactive components from plant materials [108]. Supercritical carbon dioxide (SC-CO_2_) is commonly used, reaching its critical state at 110 psi and 31.7 °C, combining the solvating power of a liquid with the diffusivity of a gas, making it highly effective for extracting natural compounds. However, SC–CO_2_’s non-polar nature limits its ability to dissolve polyphenols in grape residues. To enhance solvating power, co-solvents like water, methanol, ethanol, acetone, acetonitrile, or an acidified ethanol–water mixture are added [109,110]. This approach reduces solvent consumption, with ethanol concentrations usually ranging from 5 to 15%. SC–CO_2_ is non-flammable, non-toxic, inexpensive, and highly pure, and provides high extraction efficiency. Nevertheless, even with the use of co-solvents, SC–CO_2_ remains less effective at extracting highly polar compounds compared to techniques like PLE.

SFE is rapid, efficient, and environmentally friendly, ideal for high-quality polyphenol extraction from GP, with no residual organic solvents and controlled selectivity, making it suitable for industrial applications. Moderate extraction temperatures (around 30 °C) prevent degradation of thermally sensitive analytes. SFE’s automation and absence of air and light reduce oxidation and degradation of extracts [87].

In a study by Pazir et al. (2021) [55], SC–CO_2_ extraction with ethyl alcohol as a co-solvent efficiently extracted anthocyanins from red GP, yielding 36%, with lower environmental impact and faster processing than traditional methods. This underscores SFE’s advantages in speed and sustainability for industrial use. Similarly, Hayrapetyan et al. (2023) [109] used SC–CO_2_ with water as a co-solvent to extract polyphenols and pectic substances from GP, achieving high yields of catechin and epicatechin while preserving heat-sensitive compounds. Zhabayeva et al. (2021) [111] further demonstrated the potential of SC–CO_2_ for resveratrol extraction from GP, optimizing conditions to yield high-purity resveratrol. After the successful application of SFE, resveratrol was further purified using column chromatography with a 30% ethanol solution as the eluent, achieving enhanced purity while maintaining minimal solvent use and environmental impact. These examples highlight SFE’s precision and effectiveness in industrial-scale extraction with minimal degradation of bioactive compounds

### 4.6. Enzyme-Assisted Extraction

Enzyme-based extraction is an effective, environmentally friendly alternative to conventional methods for extracting bioactive compounds and oils. This technique targets plant cell wall components with specific enzymes, causing disruption or degradation and releasing phenolic compounds bound or present in cell vacuoles [112]. This green technology enhances bioactive compound yields and increases the bioactivity of GP extracts, recovering pharmacologically significant compounds.

Commonly used enzymes include cellulases, tannases, pectinases, glucoamylases, and proteases like trypsin and chymotrypsin [113]. These enzymes break down complex cell wall structures, releasing valuable bioactive substances. Enzyme-assisted extraction requires precise control of time, temperature, and pH due to enzyme sensitivity. Despite this challenge, the method’s benefits in yield improvement and enhanced bioactivity make it promising for industrial applications [114].

A study by Ferri et al. (2023) [115] demonstrated enzyme-assisted extraction’s efficacy for recovering phytochemicals from GP. The study found that enzyme combinations significantly improved yield and antioxidant capacity. Meini et al. (2019) [116] optimized enzymatic extraction for phenolic antioxidants from Syrah GP, enhancing phenolic yields by up to 66% and antioxidant capacity by up to 80%. Tannase raised antioxidant capacity by liberating gallic acid, while cellulase favored releasing p-coumaric acid and malvidin-3-O-glucoside.

### 4.7. Natural Deep Eutectic Solvents Extraction

Natural Deep Eutectic Solvents (NaDES) represent a novel class of solvents formed by the combination of two or more immiscible solids in specific molar ratios. When heated to a certain temperature, the eutectic point, these mixtures behave like liquids at room temperature. NaDES were initially proposed by Choi et al. (2011) [117]. The authors observed that all living organisms, whether unicellular or multicellular, consistently contain significant amounts of simple molecules such as sugars, amino acids, choline, and certain organic acids. This insight led to a new understanding of the roles these compounds play in cellular and organismal functions.

Creating an NaDES system requires a hydrogen bond donor (HBD) and a hydrogen bond acceptor (HBA). Common HBAs include amino acids and quaternary ammonium salts like choline chloride, while common HBDs are sugars and organic acids (e.g., malic, citric, lactic, succinic, tartaric acids), glucose, and glycerol, as well as urea. These components form a network of hydrogen-bond-interconnected molecules with unique physicochemical properties that can dissolve compounds of different polarities. NaDES can be highly viscous, hindering diffusion or flow, but research into their chemical structure and the use of additives like water can mitigate this issue [118,119]. However, excessive water can disrupt the solvent system, underscoring the need to optimize the molar ratio of components.

NaDES offer advantages, including their natural origin, low toxicity, biodegradability, and high solubility for various compounds [120]. Their cost effectiveness and simplicity of preparation make them attractive for large-scale industrial applications. NaDES’s potential to eliminate the need for solvent retrieval, as they can remain in consumable end products, is a significant benefit.

Recent studies have highlighted NaDES’s efficacy in extracting phytochemicals from wine by-products rich in residual bioactive polyphenols. NaDES are highly efficient in extracting thermally unstable anthocyanins [11]. Panić et al. (2019) [121] successfully scaled up NaDES-assisted extraction of anthocyanins from GP, performing extractions in half-liter batches with simultaneous multimode MAE and UAE. Their study analyzed eight NaDES combinations, finding choline chloride–citric acid (2:1) with 30% water to yield the highest total anthocyanin content. Efficient NaDES recycling was demonstrated, with a recycling yield for citric acid of 77.91% and anthocyanin recovery of about 90%.

Studies describe that NaDES enhance the antioxidant and biological properties of extracts compared to conventional solvents [122,123,124,125]. Radošević et al. (2016) [126] investigated the cytotoxic effects of choline chloride-based NaDES phenolic extracts from grape skins on MCF-7 (breast cancer) and HeLa (cervical cancer) cell lines. Among the NaDES tested, the choline chloride–malic acid (ChMa) mixture showed significant antiproliferative activity, reducing cell viability by approximately 20% at the highest concentration tested (2000 mg/L). Results suggest NaDES and their components may possess intrinsic biological activities contributing to antiproliferative effects. Malic acid, a ChMa component, has known anti-inflammatory, antioxidant, and cardioprotective properties.

In their study, Punzo et al. (2021) [127] optimized and validated sustainable extraction methods for polyphenols from GP using NaDES for cosmeceutical applications. They focused on three NaDES formulations: betaine with citric acid (BET-CA), urea (BET-U), and ethylene glycol (BET-EG). BET-CA showed the best skin permeation and was further investigated for its antioxidant and anti-inflammatory effects in human 3D keratinocytes. This formulation exhibited significant intracellular antioxidant activity, reduced the release of pro-inflammatory cytokine interleukin-8 (IL-8), and improved cell viability. Additionally, BET-CA mitigated the cytotoxic effects of menadione in human keratinocyte (HaCat) cell spheroids, indicating its potential in protecting skin cells from harmful agents.

### 4.8. Combined Techniques

Recent studies have highlighted the benefits of integrating different technologies for extracting bioactive compounds from GP, showcasing enhanced extraction yields and efficiencies.

For instance, Romanini et al. (2021) [64] found that UAE produced extracts with higher total phenolics, anthocyanins, and flavonols compared to conventional aqueous extraction. Alibante et al. (2021) [128] developed a green extraction process using ultrasonication as a pretreatment, followed by extraction with aqueous β-cyclodextrin (β-CD), resulting in extracts significantly enriched in catechin and quercetin with increased antiradical activity. Similarly, Da Porto et al. (2015) [129] found that combining UAE with SFE improved polyphenol yields and antioxidant effects.

Dranca and Oroian (2019) [130] investigated the use of SLE with various solvents, followed by an optimized UAE step, significantly increasing the extraction yield for total polyphenols and anthocyanins. Cascaes Teles et al. (2021) [131] demonstrated that enzyme-assisted extraction combined with high hydrostatic pressure significantly boosted enzyme activity, making the process more efficient and economical compared to traditional methods.

Marianne et al. (2024) [132] optimized a green extraction process using ultrasonication combined with MAE for GP, yielding extracts with significantly higher concentrations of phenolic compounds and antioxidant activity compared to conventional methods. Finally, Neto et al. (2021) [120] found that combining NaDES with MAE was particularly effective for extracting proanthocyanidins, achieving high yields in a short time.

Integrating advanced extraction techniques with conventional methods significantly enhances the efficiency and yield of bioactive compound extraction from GP. This multifaceted approach not only improves the economic feasibility of the extraction processes but also promotes sustainability by utilizing greener solvents and reducing extraction times.

Table 2 provides an overview of several studies conducted to extract phenolic compounds from GP using various extraction techniques. Total Phenolic Content (TPC), commonly measured by the Folin–Ciocalteu method, is often used to estimate overall phenolic levels due to its simplicity. However, TPC does not provide precise information on specific phenolic compounds and may lead to overestimation if certain phenolics react more strongly with the reagent. A more detailed discussion on the spectrophotometric methods for estimating phenolic compounds and antioxidant capacity can be found in Section 6.1.

### 4.9. Post-Extraction Purification and Isolation of Phenolic Compounds

Following the application of various extraction methods, additional steps are often necessary to further refine the recovered phenolic compounds. Techniques such as liquid–liquid extraction or solid-phase extraction (SPE) are commonly employed to purify and isolate the desired polyphenolic fractions from the crude extract [47,101]. SPE is particularly effective at removing impurities and concentrating specific phenolic compounds, ensuring cleaner fractions for subsequent analysis. Other methods like column chromatography, gel filtration, and ion-exchange chromatography may also be utilized, depending on the nature of the phenolic compounds and matrix. The use of these additional purification steps is crucial not only for enhancing the yield and purity of the isolated phenolics but also to ensure their accuracy and reliability in downstream chemical analyses, which often require precise quantification and characterization of individual compounds.

## 5. Analysis of Grape Pomace Polyphenols

Polyphenols are naturally occurring compounds found abundantly in a variety of plants, although usually in small amounts. Given their numerous health benefits and wide-ranging applications in functional foods, nutraceuticals, and pharmaceuticals, they have attracted significant interest [136]. This has led to the necessity for precise and sensitive analytical methods for their detection. In recent decades, advanced techniques such as capillary electrophoresis, gas chromatography, and high-performance liquid chromatography with various detectors have been employed for the analysis of polyphenols in plant matrices.

Capillary electrophoresis (CE) is an analytical technique suitable for separating small molecules and distinguishing between isomers. CE offers several advantages, including rapid and accurate analysis with minimal sample and reagent usage [137]. This method works by applying an electric field to a capillary filled with a buffer solution, causing charged molecules to migrate at different rates based on their size and charge. CE has been effectively used for analyzing polyphenols, but its application in GP analysis is less documented. For example, Rockenbach et al. (2012) [138] used CE to identify polyphenols in grape seed extracts, highlighting its capability to detect enantiomers of catechin and epicatechin. They found that the fermentation process did not produce (−)-catechin and (+)-epicatechin enantiomers; only (+)-catechin and (−)-epicatechin were detected.

Gas chromatography–mass spectrometry (GC-MS) is a highly sensitive and accurate analytical technique capable of achieving very low detection limits. Techniques like pyrolysis GC-MS are particularly useful for analyzing high-molecular-weight compounds like procyanidins due to the large number of fragments produced [87]. However, GC-MS poses challenges for polyphenol analysis because polyphenols are non-volatile, requiring a derivatization step. Additionally, GC-MS can cause isomerization and degradation of analytes, making it less practical. GC-MS has occasionally been used to analyze phenolic compounds in grapes and wine. The analysis of phenolic compounds from GP using GC-MS has not been commonly discussed in the recent literature; instead, it focuses exclusively on the volatile components [139,140,141] or fatty acids [74,142].

High-performance liquid chromatography (HPLC) is widely used for the analysis of phenolic compounds in grapes and GP due to its reliability, high sensitivity, robustness, and ability to produce accurate results in complex matrices [84]. HPLC can be paired with different detection systems, such as UV–Visible, diode array, or fluorescence detectors, to accurately and sensitively detect compounds. Typical wavelengths for measuring phenolic compounds include 280 nm for flavonoids and 330 nm and 520 nm for anthocyanins [143]. Numerous studies using Reverse-Phase HPLC systems with a diode array detector (DAD) have been conducted for the determination of the phenolic profile of GP [65,125,144,145]. However, HPLC combined with mass spectrometry (MS) has emerged as the best method for identifying phenolic compounds. MS is renowned for its versatility and sensitivity in the structural elucidation and profiling of phenolic compounds and is capable of detecting analytes at very low concentrations. It offers high specificity and selectivity by measuring the mass-to-charge ratio (*m*/*z*) of ions, enabling precise identification of compounds in complex matrices. Additionally, MS provides accurate quantification of analytes by distinguishing them from background noise and co-eluting compounds, overcoming the limitations of UV–Visible detectors [87].

The main features of HPLC-MS include high sensitivity, selectivity, speed, and a wide dynamic range. Various mass spectrometers, including low- and high-resolution types, are available. Low-resolution detectors such as ion trap (IT) or triple quadrupole (QqQ) detectors are commonly used, with LC-QqQ being the most common technique for targeted screening of phenolic compounds in GP [41,146,147].

While low-resolution MS remains useful for identifying and quantifying polyphenols in various matrices, high-resolution mass spectrometry (HRMS), such as quadrupole time-of-flight spectrometry (Q-ToF) and quadrupole-Orbitrap technologies, has become more popular [84]. HRMS provides detailed information on the exact molecular mass, elemental composition, and chemical structure of a compound with a mass error of ≤2 ppm, making it ideal for structural characterization of polyphenols.

Onache et al. (2022) [13] used Q-Orbitrap HRMS to analyze the phenolic content in different white and red GP samples. The high-resolution capabilities of the Q-Orbitrap allowed for accurate mass determination and structural elucidation of 26 polyphenolic compounds, including phenolic acids, flavonoids, and stilbenes. This analysis facilitated the identification of minor metabolites that are challenging to resolve using low-resolution techniques. Similarly, another study investigated the phenolic compounds in GP using LC-QToF-MS. The findings highlighted the efficacy of this high-resolution technique in profiling and quantifying polyphenols, providing essential data on the phenolic composition and antioxidant potential of the GP extracts. The study underscored the importance of HRMS in offering the precise and comprehensive chemical characterization essential for understanding the bioactive properties of polyphenols [10].

Moreover, the integration of Matrix-Assisted Laser Desorption/Ionization Time-of-Flight Mass Spectrometry (MALDI-ToF-MS) with other high-resolution techniques further enhances the analytical depth and accuracy, making it indispensable in modern analytical chemistry. MALDI-ToF-MS has proven to be a valuable tool for the characterization of complex mixtures and the analysis of high-molecular-weight compounds and polymers [148].

As documented by Oliveira et al. (2015) [58], MALDI-ToF-MS and LC-DAD/MS were utilized to analyze anthocyanins in GP. This dual approach allowed for the detection of over 50 anthocyanin-based pigments, including pyranoanthocyanins and oligomeric malvidin-3-O-coumaroylglucoside-based anthocyanins. MALDI-ToF-MS was particularly effective in this analysis due to its ability to produce singly charged molecular ions for each parent molecule, thereby avoiding the ion stacking issues that can complicate other mass spectrometric techniques.

In another study, Pérez-Ramírez et al. (2018) [149] conducted a comprehensive characterization of both extractable and non-extractable phenolic compounds in a grape/pomegranate pomace dietary supplement, combining two analytical techniques. The use of HPLC-ESI-QToF MS/MS allowed for the identification of specific phenolic compounds through their unique mass spectrometric fragmentation patterns, while MALDI-ToF-MS provided insights into high-molecular-weight polymeric phenolic structures that are not easily separated by conventional liquid chromatography techniques. The study revealed a detailed profile of phenolic compounds, including several anthocyanins, gallotannins, and gallagyl derivatives, many of which were reported in grape or pomegranate for the first time.

Collectively, these studies illustrate the pivotal role of high-resolution mass spectrometry in advancing our understanding of the polyphenolic composition in GP.

## 6. Grape Pomace Polyphenols’ Health Benefits

### 6.1. Antioxidant Properties

Oxidative stress arises from an imbalance between oxidants and antioxidants, primarily due to either a deficiency in antioxidant defenses or an overproduction of free radicals. Free radicals, characterized by an unpaired electron in their outer orbit, are highly unstable and reactive molecules. Their formation can be triggered by external factors such as heat, ultraviolet irradiation, and air pollution, or naturally within mitochondria. Reactive species, including reactive oxygen species (ROS), reactive nitrogen species (RNS), and reactive sulfur species (RSS), contribute to this imbalance. The excess of free radicals can lead to significant biological damage, impacting various cellular components and contributing to diseases such as cancer, type 2 diabetes, Alzheimer’s, and cardiovascular diseases [150,151,152].

Antioxidants are substances that mitigate oxidative damage to biological molecules by scavenging free radicals, interrupting radical-initiated chain reactions, and repairing or eliminating damaged structures. They originate from both endogenous sources (enzymatic and non-enzymatic) and dietary sources, working together synergistically to maintain or restore redox homeostasis [153,154,155].

GP contains high amounts of polyphenols, known for their health-promoting properties. Polyphenols, natural antioxidants found in plants, have been widely studied for their potential health benefits, including reducing the risk of chronic diseases, combating oxidative stress, and exhibiting anti-inflammatory and anti-carcinogenic effects [17,156].

Polyphenols neutralize free radicals by transferring electrons or hydrogen atoms, which stabilizes their own molecular structure and enhances their antioxidant effectiveness. This stabilization is due to the resonance and delocalization of the unpaired electron across the molecule, making the radical form less reactive. Additionally, the hydroxyl groups in polyphenols can form intramolecular hydrogen bonds, further increasing stability [157,158].

The antioxidant capacity of polyphenols is largely attributed to their chemical structure, specifically, the number and position of hydroxyl groups. These structural features enable polyphenols to act as free radical scavengers, hydrogen donors, metal chelators, and quenchers of singlet oxygen. Additionally, they inhibit lipid oxidation and reduce peroxide formation [19,36,159]. Beyond these actions, polyphenols modulate endogenous pathways to combat oxidative stress by activating nuclear factor E2 and up-regulating enzymes such as superoxide dismutase (SOD), catalase (CAT), glutathione, glutathione peroxidase (GPx), and heme-oxygenase [160]. These activities help prevent oxidative damage in cells, reduce the risk of chronic diseases, and promote overall health.

Research consistently highlights the significant antioxidant capabilities of GP. Chedea et al. (2019) [161] (Table 3) studied the effects of a 5% dried GP diet in piglets over 36 days. The study compared a control group with an experimental group receiving the GP diet. The results revealed that the total antioxidant status in the liver increased by 21%, in the spleen by 20%, and in the kidneys by 11%. Antioxidant enzyme activities were enhanced, with CAT activity increasing by 13% in the spleen and 18% in the kidneys, and SOD activity increasing by 11% in the liver, 21% in the spleen, and 13% in the kidneys. GPx activity remained unchanged in the liver and spleen but increased significantly by 33% in the kidneys. Lipid peroxidation, measured by thiobarbituric acid reactive substances (TBARS)-malondialdehyde (MDA) levels, decreased significantly in the liver by 34% and in the kidneys by 30%, indicating reduced oxidative stress in these organs. The total polyphenol content in the blood and various organs was higher in the GP-fed piglets after 36 days, suggesting effective absorption and distribution of dietary polyphenols. The study concluded that incorporating 5% dried GP into the diet of piglets improves the antioxidant status of key organs, thereby contributing to better overall health by enhancing antioxidant defenses and reducing oxidative stress markers.

The usefulness of GP’s natural antioxidants for medical purposes was also mentioned in another study [162] (Table 3). Using both in vitro and in vivo models, specifically, the nematode *Caenorhabditis elegans*, the researchers assessed the antioxidant qualities and biological effects of four GP extracts high in polyphenols. The study measured the total phenolic content and antioxidant activities of the GP extracts. Different concentrations of GP extracts were tested on *C. elegans* under thermally induced oxidative stress, revealing that low concentrations increased the worms’ stress resistance, while higher concentrations had detrimental effects. Low concentrations generally reduced ROS levels, correlating with increased resistance to oxidative stress, whereas higher concentrations had mixed effects. The most active extract, L3, increased the maximum lifespan of *C. elegans* but did not significantly extend the mean lifespan, indicating a hormetic response where low doses are beneficial but high doses are harmful. The extracts, rich in phenolic acids like gallic acid and ellagic acid, along with flavan-3-ols and flavonols, demonstrated significant antioxidant properties.

Annunziata et al. (2021) [163] (Table 3) investigated the antioxidant activity of Taurisolo^®^, a polyphenolic extract from Aglianico GP, on human neutrophils from subjects with metabolic syndrome. The study aimed to assess its efficacy in reducing oxidative stress markers and modulating antioxidant enzyme activities. Both native and digested forms of the extract exhibited significant antioxidant activity. Despite a slight decrease after gastrointestinal digestion, the antioxidant capacity remained substantial. Taurisolo^®^ significantly reduced ROS levels in neutrophils, particularly when oxidative stress was induced by phorbol 12-myristate 13-acetate (PMA).

Taurisolo^®^ regulated the activities of CAT and myeloperoxidase (MPO), two key antioxidant enzymes. While PMA increased the activities of CAT and MPO in the extracellular media, Taurisolo^®^ significantly decreased these activities. Conversely, in the intracellular media, Taurisolo^®^ treatment increased CAT and MPO activities, suggesting an up-regulation of the antioxidant defense system. The extract also reduced MDA levels, a marker of lipid peroxidation, indicating its role in preventing oxidative damage to cell membranes. Additionally, it influenced the expression of oxidative-stress- and inflammation-related genes. It down-regulated cyclooxygenase-2 (COX-2) and tumor necrosis factor α (TNF-α) (pro-inflammatory genes) and up-regulated interleukin-10 (IL-10) (an anti-inflammatory gene).

The measurement of antioxidant activity in GP is most commonly performed using spectrophotometric methods, which involve colorimetric techniques to detect changes in color that correspond to antioxidant levels. For example, the Folin–Ciocalteu test measures TPC by the reduction of a reagent under alkaline conditions. However, one important problem is that it can be non-specific and react with other compounds. The ABTS (22′-Azinobis(3-Ethylbenzothiazoline-6-Sulfonic Acid) test measures the reduction of a radical cation and is quick and can be performed over a wide pH range, though the radical generation process is complex. The DPPH (22-Diphenyl-1-Picrylhydrazyl) test measures antioxidant capacity based on the reduction of a stable radical and is popular due to its simplicity and speed. The FRAP (Ferric-Reducing Antioxidant Power) test measures the reducing power of antioxidants by their ability to reduce ferric ions to ferrous ions and is fast and cost effective, though it does not directly measure radical-scavenging capacity [164].

However, spectrophotometric methods often result in inaccurate estimates of polyphenol content and antioxidant capacity due to interference from other molecules. Additionally, as Xu et al. (2015) [165] indicated, each test may be more specific in detecting certain compounds over others. Consequently, it is crucial to combine multiple procedures to obtain accurate results as these methods alone are not very reliable.

To overcome some of the limitations of spectrophotometric methods, electrochemical methods, such as chronoamperometry, differential pulse voltammetry, and cyclic voltammetry, offer a faster, more robust alternative for quantifying polyphenols and determining antioxidant capacity. These methods analyze the electrochemical activity of phenolic compounds, allowing for the differentiation between various types of molecules based on their oxidation potentials [166]. Cyclic voltammetry, in particular, is the most commonly used electrochemical method for this purpose, providing detailed analysis of the antioxidant capacity of the sample. Studies like that by Vasyliev et al. (2020) [167] have demonstrated the application of cyclic voltammetry to evaluate the reducing ability and antioxidant activity of GP. This method has shown good correlation with traditional chemical techniques, confirming its reliability.

Their research evaluated the redox behavior of GP using cyclic voltammetry alongside traditional methods like the FRAP and phosphomolybdenum assays. They found that the reducing power of grape extract was higher than that of apricot and black currant extracts. Cyclic voltammetry provided detailed insights into the redox characteristics, showing that GP had a strong reducing capacity, which correlated well with its antioxidant activity.

Jara-Palacios et al. (2014) [168] investigated the antioxidant potential of white GP using cyclic voltammetry, discovering that it significantly reduced ROS levels in human colonic epithelial cells exposed to hydrogen peroxide (H_2_O_2_)-induced oxidative damage. This finding underscores the potential of GP in mitigating oxidative stress, thus supporting its application in health-related fields. In a subsequent study conducted in 2017, the same research team demonstrated that cyclic voltammetry is an effective method for measuring the total antioxidant potential of GP and its components (seeds, skins, and stems). They found that pomace and seeds exhibited the highest voltammetric peak areas, indicating greater antioxidant potential compared to skins and stems. Furthermore, a significant correlation was observed between the voltammetric parameters and the inhibition of lipid peroxidation, suggesting that cyclic voltammetry is a promising technique for assessing the antioxidant capacity of phenolic extracts from winemaking by-products [169].

Finally, some studies also report the use of cyclic voltammetry as a useful technique for determining the antioxidant and prooxidant properties of GP extracts [170,171].

### 6.2. Anti-Inflammatory Properties

Inflammation is the immune system’s response to foreign pathogens, physical damage, ultraviolet radiation, and microbial invasion, aimed at eliminating harmful stimuli and initiating tissue healing. Key physical signs include pain, heat, redness, and swelling, resulting from increased blood flow, vasodilation, release of intracellular mediators, and fluid leakage [172,173].

Inflammation is categorized in two types, acute and chronic. Acute inflammation is a swift response that initiates within minutes of tissue injury, aiming to eliminate bacteria, viruses, or parasites at the infection site. This process involves the action of plasma proteins and the movement of fluids and neutrophils to the affected area, primarily activating neutrophils and macrophages. In contrast, chronic inflammation is a prolonged process characterized by vascular proliferation, the accumulation of macrophages, fibrosis, and tissue destruction. The primary agents of chronic inflammation are T-lymphocytes, plasma cells, and macrophages [173,174,175].

Macrophages play a central role in inflammation, secreting pro-inflammatory cytokines such as TNF-α, interleukin-6 (IL-6), and interleukin-1 beta (IL-1β), along with pro-inflammatory mediators like nitric oxide (NO), prostaglandin E2 (PGE2), and ROS. They also produce inflammatory proteins like inducible nitric oxide synthase (iNOS) and COX-2. Controlling inflammation involves inhibiting these inflammatory markers [176,177,178].

Chronic inflammation can damage healthy tissues, leading to diseases such as asthma, aging, atherosclerosis, AIDS, gout, diabetes, Alzheimer’s disease, Parkinson’s disease, cancer, and heart failure [179,180]. While non-steroidal anti-inflammatory drugs and corticosteroids are commonly used to treat inflammatory disorders, they can cause adverse effects like hypertension, hyperglycemia, muscle weakness, osteoporosis, and diabetes [181]. Research suggests that phytochemicals, such as polyphenols, possess anti-inflammatory properties and may serve as alternatives to synthetic chemicals [182].

Polyphenols, found abundantly in various fruits and vegetables, exhibit strong anti-inflammatory properties. These natural compounds inhibit key inflammatory pathways, such as the mitogen-activated protein kinases (MAPKs) pathway and nuclear factor kappa B (NF-κB) pathway, leading to a reduction in the production of inflammatory molecules like cytokines and chemokines. Additionally, polyphenols block cyclooxygenase and lipoxygenase enzymes, which are involved in creating inflammatory compounds like prostaglandins, thromboxane A2, and leukotrienes [160].

Berry-derived polyphenols, such as those from bilberries, black raspberries, blueberries, and grapes, play a significant role in reducing inflammation. They lower levels of inflammatory markers like inhibitor of nuclear factor kappa B (phospho-IκBα), COX-2, and PGE2, providing relief in conditions like colitis [183]. Blueberry (*Vaccinium corymbosum*) anthocyanins, in particular, decrease the expression of pro-inflammatory mediators such as TNF-α and IL-1β and reduce oxidative stress in hypercholesterolemic conditions [184]. Moreover, polyphenols from other sources, such as plums (*Prunus salicina*), have shown significant anti-inflammatory effects. Studies have demonstrated that these polyphenols can inhibit inflammatory factors like TNF-α, IL-1β, and interleukin-18 (IL-18), enhance the activity of SOD, and reduce levels of intracellular ROS and MDA. These effects are achieved through mechanisms involving the hypoxia-inducible factor 1 (HIF-1), erythroblastic leukemia viral oncogene homolog B (ErbB), and forkhead box O (FoxO) signaling pathways, which play crucial roles in cellular stress responses and inflammation [185].

Additionally, a study on polyphenols from grape berry skins, specifically Merlot, Tannat, and Syrah varieties, demonstrated significant anti-inflammatory potential. These grape skin extracts inhibited NO and ROS production in lipopolysaccharide (LPS)-stimulated macrophages. This study highlighted that grape skin extracts could effectively reduce inflammation and oxidative stress, supporting their use as natural anti-inflammatory agents [186].

Concerning GP polyphenols, they are effective in reducing inflammation by lowering mitochondrial ROS and inflammatory markers like TNF-α, IL-1β, IL-6, and NF-κB. These polyphenols’ actions on various inflammatory pathways highlight their potential as natural anti-inflammatory agents [17]. Research highlights the beneficial in vitro and in vivo effects of different red and white GP in inflammatory conditions. For instance, Recinella et al. (2022) [187] (Table 3) proved that a water extract of GP modulated the inflammatory and immune response in the human colorectal cancer cell line SW480 and in an isolated mouse colon exposed to *Escherichia coli* LPS. The extract decreased cell viability, mRNA levels of NF-κB, COX-2, TNF-α, IL-6, IL-1β, IL-10, iNOS, and interferon gamma (IFNγ) in the isolated colon, and increased gene expression of antioxidant enzymes such as CAT and SOD. Calabriso et al. (2022) [145] (Table 3) reported the attenuation of the inflammatory response in human endothelial cells by reducing the expression of adhesion molecules and pro-inflammatory cytokines. The study showed that the extract inhibited the adhesion of monocytes to endothelial cells and decreased the levels of TNF-α and IL-6, suggesting its potential in reducing vascular inflammation. Abbasi-Parizad et al. (2021) [188] (Table 3) conducted a study on the recovery of phenolic compounds from agro-industrial grape wastes and their anti-inflammatory activity. In this regard, it was established that phenolic-rich extracts from GP significantly suppressed the production of cytokines and mediators of inflammation, including TNF-α, IL-1β, and COX-2, potentially natural anti-inflammatory agents. These examples underscore the potential of GP polyphenols as natural agents for the management of inflammation, hence offering a promising alternative to conventional anti-inflammatory drugs.

### 6.3. Metabolism of Grape Pomace Polyphenols

The metabolism of bioactive compounds is a critical determinant of their efficacy and biological impact on human health. Upon ingestion, these compounds undergo a series of biochemical processes—collectively known as ADMET (Absorption, Distribution, Metabolism, Excretion, and Toxicity)—which influence their pharmacokinetics, bioavailability, and therapeutic applications. In the case of plant-derived bioactive compounds, the metabolic pathways involved can be quite complex, often resulting in the formation of metabolites with distinct biological activities from the original compounds. Understanding these transformations is essential for deciphering the health-promoting effects of polyphenol-rich extracts, and their long-term implications in disease prevention.

GP polyphenols, in particular, have drawn increasing attention due to the significant role their metabolism plays in defining their biological activity and therapeutic potential. The following section will explore the processes underlying the absorption, metabolism, and utilization of GP polyphenols, illustrating how these compounds contribute to various health benefits.

One of the key factors influencing the bioavailability of GP polyphenols is their absorption, which primarily occurs in the small intestine. However, this process is often hindered by their large molecular size and complex structure, limiting efficient uptake. As a result, most GP phenolics pass through the upper gastrointestinal tract and reach the colon, where microbial metabolism converts them into smaller, bioactive metabolites that frequently exhibit enhanced biological activity compared to their parent compounds [52]. In their study, Taladrid et al. (2021) [189] (Table 3) used a dynamic gastrointestinal simulator (simgi^®^) to replicate the gastrointestinal digestion of a GP extract. This simulation involved both intestinal and colonic digestion phases, producing two distinct fluids for analysis. The results showed that colonic fermentation of the GP extract significantly promoted the production of short-chain fatty acids (SCFAs) and phenolic metabolites. These metabolites played an important role in reducing intestinal permeability and demonstrated the importance of microbial-mediated metabolism in modulating the bioactivity of GP phenolics.

Ramos-Romero et al. (2021) [190] (Table 3) further highlighted the significant variability in the absorption of GP phenolics between individuals, largely driven by differences in gut microbiota composition. Individuals with favorable gut microbial profiles, characterized by lower levels of *Firmicutes* and *Prevotella*, exhibited improved insulin sensitivity and a better response to GP extract intake. This underscores the role of gut microbiota in modulating the bioavailability of GP phenolics. Moreover, GP supplementation has been shown to beneficially alter gut microbiota, increasing the presence of bacteria such as *Lactobacillus* and *Bifidobacterium* while reducing harmful species like *Clostridium* [52]. These changes in gut microbiota are vital, as they not only support gut health but also influence systemic metabolic parameters such as lipid levels and glucose tolerance.

Following absorption, GP phenolic metabolites are distributed through the bloodstream to various tissues, where they exert antioxidant and anti-inflammatory effects. For instance, Rasines-Perea et al. (2018) [191] (Table 3) investigated the effects of GP extracts from Rhône Valley red wine cultivars (Grenache, Syrah, Mourvèdre, and Alicante) on hypertensive rat models. Over six weeks, rats receiving these GP extracts experienced reduced oxidative stress, lower blood pressure, and increased antioxidant enzyme activity. These outcomes were attributed to the bioavailability of polyphenolic metabolites, such as glucuronides and sulphates, which were distributed to tissues including the liver, kidneys, and heart. The study also noted that a rebound effect in blood pressure occurred after treatment interruption, highlighting the role of continuous intake of GP extracts in managing hypertension. These findings emphasize that the metabolic conversion of polyphenols like resveratrol and proanthocyanidins is critical for their cardiovascular benefits.

In addition to cardiovascular health, the metabolism of GP polyphenols by gut microbiota significantly influences broader health outcomes, by producing bioactive metabolites, including SCFAs like acetate, propionate, and butyrate, as well as phenolic acids such as benzoic, phenylacetic, and cinnamic acids. These metabolites significantly influence metabolic health by improving insulin sensitivity, reducing blood glucose, and positively impacting lipid profiles. Moreover, they help reduce inflammation and oxidative stress by modulating key pathways like the NF-κB and nuclear factor erythroid 2–related factor 2 (Nrf2) pathways [27].

An example of this metabolic transformation is the production of phenyl-γ-valerolactones, the most abundant and bioavailable class of phenolic metabolites derived from GP polyphenols. These metabolites are formed via microbial metabolism in the colon and have been shown to persist in circulation for extended periods, peaking in plasma 4 to 10 h post ingestion and being excreted in urine up to 48 h later. In addition to phenyl-γ-valerolactones, other key metabolites such as hydroxybenzoic acids, simple phenols like methylpyrogallol-sulphate, protocatechuic acid-3-sulphate, and (epi)catechin conjugates undergo extensive phase II metabolism, resulting in sulfate and glucuronide conjugates, which remain detectable in circulation for several hours [192]. The sustained bioavailability and prolonged presence of these metabolites underscore the crucial role of gut microbial metabolism and phase II conjugation in transforming GP polyphenols into bioactive compounds. These transformations enhance their potential to support metabolic health, reduce inflammation, and counteract oxidative stress, though the exact mechanisms involved are yet to be fully understood.

The excretion of GP phenolic metabolites occurs primarily through urine and feces, completing the ADMET cycle. The ability of the body to process and utilize these metabolites is essential for maintaining homeostasis and preventing potential toxicity. Importantly, studies have shown that long-term supplementation with GP phenolics does not result in adverse effects. For example, Martínez-Maqueda et al. (2018) [28] (Table 3) showed that six weeks of GP supplementation improved insulin sensitivity without negatively impacting other cardiometabolic markers, indicating that GP phenolics can be a safe, promising intervention for metabolic health, particularly in individuals at risk of insulin resistance. However, the safety of higher doses or extended use remains uncertain and requires further research.

Overall, GP phenolics demonstrate considerable potential, particularly for their antioxidant, anti-inflammatory, and metabolic benefits. Their bioavailability, particularly of key metabolites such as phenyl-γ-valerolactones, phenolic acid, and (epi)catechin conjugates, is crucial in determining their bioactivity and health-promoting effects. The potential of GP polyphenols has already spurred the development of various patents and nutraceutical products, positioning it as an emerging ingredient in health and wellness. However, further research is crucial to fully understand their synergistic effects with other bioactive compounds and to establish the safety of higher doses or extended supplementation. While the current findings are promising, more comprehensive, long-term studies are necessary to validate their therapeutic value and ensure their safe, effective use in clinical and consumer applications.

**Table 3:** Overview of grape pomace phenolic compounds’ health benefits: dosages, biological systems, and key bioactivities.

Study	System Studied	Quantity of Grape Pomace Administered	Main Results
Martínez-Maqueda et al., 2018 [28]	Human subjects with cardiometabolic risk	8 g/day of dried GP for 6 weeks	Improved insulin sensitivity (reduction in fasting insulin), with no significant effect on other cardiometabolic risk factors.
Calabriso et al., 2022 [145]	Caco-2/HMEC-1 co-culture model (intestinal epithelial and endothelial cells)	1, 5, and 10 µg/mL of GP extract (gallic acid equivalent)	GP extract attenuated the expression of inflammatory markers such as IL-6 and TNF-α in a concentration-dependent manner. It also reduced endothelial cell adhesion molecule expression (VCAM-1, ICAM-1) and leukocyte adhesion under pro-inflammatory conditions.
Chedea et al., 2019 [161]	Piglets (TOPIG hybrid)	5% dried GP in feed for 36 days	Significant increase in antioxidant enzyme activity (SOD, CAT, GPx) in the liver, spleen, and kidneys, along with improved total antioxidant status and reduced lipid peroxidation in key organs. No significant effects on body weight or feed-to-gain ratio.
Ayuda-Durán et al., 2019 [162]	In vivo model using *Caenorhabditis elegans*	100 to 1000 µg/mL of GP extract	GP extracts rich in polyphenols increased lifespan and stress resistance in *C. elegans* at lower concentrations, with hormetic effects observed at higher concentrations. Improved resistance to thermally induced oxidative stress and decreased ROS accumulation.
Annunziata et al., 2021 [163]	Ex vivo study on human neutrophils from subjects with metabolic syndrome	1 mg/mL of Taurisolo^®^ (GP extract)	Taurisolo^®^ significantly reduced oxidative stress markers (ROS levels) and inflammatory cytokines (COX-2, TNF-α) while enhancing intracellular antioxidant enzyme activity (CAT, MPO). Reduced MDA levels indicate protection against lipid peroxidation.
Recinella et al., 2022 [187]	SW-480 human colorectal cancer cells and isolated mouse colon	1–1000 µg/mL of GP water extract	Significant reduction in SW-480 cell viability, downregulation of pro-inflammatory markers (NF-κB, COX-2, TNF-α, IL-6), increase in BAX/BCL-2 proteins ratio indicating apoptosis, and modulation of antioxidant and inflammatory response in mouse colon.
Abbasi-Parizad et al., 2021 [188]	In vitro study using Caco-2 cells	15 µg/mL and 25 µg/mL of GP extract	GP extract demonstrated significant anti-inflammatory properties by inhibiting IL-8 expression, with a dose-dependent response. The higher concentration (25 µg/mL) resulted in 85.6% inhibition of cytokine IL-8 expression.
Taladrid et al., 2021 [189]	In vitro Caco-2 cell model of intestinal barrier	1:40 diluted GP extract	GP extract reduced paracellular permeability by enhancing tight junction proteins, such as ZO-1 and occludin. Colonic digested extracts also showed protective effects on gut permeability.
Ramos-Romero et al., 2021 [190]	Human subjects with cardiometabolic risk	8 g/day of dried GP for 6 weeks	Responders exhibited lower *Firmicutes* and *Prevotella* levels and higher microRNA-222 levels, suggesting impaired glycaemic control improvement. Responders showed better insulin sensitivity while non-responders had no significant changes.
Rasines-Perea et al., 2018 [191]	Spontaneously hypertensive rats (SHR)	21 mg/kg/day of GP extract for 6 weeks	GP extract showed a “rebound effect” on systolic blood pressure, particularly in Grenache seed extract, Syrah seed extract and Alicante skin extract groups, suggesting a potential antihypertensive effect.
Castello et al., 2018 [192]	Human subjects (10 healthy volunteers)	250 mL of red grape pomace drink (625 mg/100 mL of total polyphenols)	Phenyl-γ-valerolactones were the most abundant and bioavailable metabolites, peaking 4–10 h post ingestion. (Epi)catechin conjugates and hydroxybenzoic acids were also bioavailable, though cleared more quickly. Metabolites remained detectable in plasma for several hours and in urine for up to 48 h. Significant inter-individual variability was observed in both plasma and urinary metabolite levels.

## 7. Conclusions and Future Perspectives

The valorization of by-products has emerged as a central focus in modern scientific research. By converting waste into valuable resources, industries can reduce their environmental impact while enhancing cost efficiency. This approach aligns with the global movement towards a circular economy, where waste is minimized and resources are reused or repurposing in innovative ways. Particularly considering the rapidly growing global population, the demand for natural, health-focused, and sustainable solutions has never been greater.

This review has thoroughly examined the phenolic composition of GP, highlighting its therapeutic potential and significant health benefits, which establish a strong foundation for future research. GP’s unique composition, rich in polyphenols and other bioactive compounds, transforms it from a winemaking by-product into a versatile and valuable resource with potent antioxidant and anti-inflammatory properties. These findings position GP as a promising candidate for various applications and highlight its potential to be developed into sustainable, economically viable, high-value products across multiple industries.

In the food industry, the potential of GP is increasingly being recognized. Its high fiber and polyphenol content not only enhance the nutritional value of food products but also improve their technological properties. Current research highlights the potential of GP extracts as natural antioxidants, which not only extend the shelf life of foods but also reduce the need for synthetic additives. This shift toward natural preservation methods aligns with consumer preferences for clean-label and eco-friendly products. GP’s ability to prevent lipid oxidation in foods like meats and oils further demonstrates its value in food preservation, offering a more sustainable alternative to traditional methods. Recent patents also underscore its versatility, highlighting its use in a wide range of formulations, from baked goods to dairy products and beverages.

Beyond its applications in food, GP holds significant potential in the nutraceutical and dietary supplement industries. The bioactive compounds present in GP, such as polyphenols, have been linked to promoting metabolic health, reducing oxidative stress, and potentially preventing chronic diseases like cardiovascular conditions. As the demand for natural, health-promoting supplements grows, GP’s therapeutic potential could make it a key ingredient in future product development. This is a promising area for ongoing research, with considerable potential to expand GP’s role in preventive health strategies.

However, a key challenge remains in scaling up GP extraction processes to ensure their impact across industries. Advances in extraction technologies are essential to maintain the purity and efficacy of GP polyphenols while being cost effective and environmentally sustainable. Currently, research predominantly focuses on the biological effects of whole GP extracts, with limited exploration of individual phenolic families within these extracts. Investigating the biological actions of purified phenolic compounds and their synergistic effects could lead to more targeted applications in nutraceuticals and pharmaceuticals and enhance therapeutic benefits. Furthermore, given the growing interest in functional foods and personalized nutrition, such research could play a pivotal role in the development of precise dietary interventions aimed at preventing chronic diseases.

In the cosmetics industry, recent regulatory changes in the European Union, including restrictions on certain antioxidants in skincare products, are creating opportunities for natural alternatives such as phenolic compounds derived from GP. Its potent antioxidant and anti-inflammatory properties make GP extracts a promising ingredient for skincare products and topical formulations. Although their current use in cosmetics is limited, the growing demand for eco-friendly and sustainable products could drive further interest in GP as a natural, safe, and effective alternative in this sector.

In summary, GP represents an invaluable resource with a wide range of current and potential applications in both the health and food industries. Its rich polyphenolic content offers natural solutions as antioxidant agents or as active ingredients, aligning with global sustainability goals. Further exploration of GP’s potential will not only leverage its health benefits but also promote sustainability within the winemaking industry. To fully unlock the potential of GP, further research and innovation are essential. By refining extraction techniques and exploring new applications in emerging markets like cosmetics and pharmaceuticals, the value of this remarkable by-product could be maximized. GP stands as a testament to how waste can be transformed into a resource, offering substantial environmental, economic, and health benefits.

## Figures and Tables

**Table 2:** Different methods used for the extraction of polyphenols from grape pomace.

Extraction Method	Raw Material	Main Compounds Identified	Phenolic Content	Reference
UAE (Ethanol, 25 kHz, 20 °C, 60 min)	Agiorgitiko red GP	Delphinidin, Malvidin-3-O-glucoside, Myricetin, Quercetin	TPC: 438,984 ± 4034 ppm GAE	Drosou et al., 2015 [133]
UAE (Temperature: 80 °C, Time: 4 min) and SFE	Red GP	Proanthocyanidins	TPC: 3493 ± 61 mg GAE/100 g DW	Da Porto et al., 2015 [129]
UAE (Frequency: 2458 MHz, Power Density: 1000 W/L, Time: 30 min, Temperature: 28 ± 3 °C)	Red skin GP	Malvidin-3-O-glucoside, Quercetin, Catechin, Myricetin	TPC: 104 mg GAE/g	Caldas et al., 2018 [134]
MAE (Time: 10 min, Power: 93 W, Temperature: 24 ± 1 °C)	Chardonnay GP	Flavanols, Quercetin, Kaempferol, Gallic acid	TPC: 1.21 ± 0.04 mg GAE/mL	Garrido et al., 2019 [62]
Subcritical Water Extraction (SWE) (Time: 10 min, Pressure: 10.34 MPa) and NaDES	Red GP	Catechin, Epicatechin, Procyanidins, Resveratrol	Catechin and epicatechin: 45.05% and 47.98% increase	Loarce et al., 2020 [44]
SLE (Ethanol, Ethanol –Water, Water)	White GP (whole, skins, seeds, stems)	Gallic acid, Catechin, Quercetin-3-O-glucoside, Kaempferol-3-O-glucoside	TPC: 2797.67–9839.86 mg GAE/100 g	Jara-Palacios et al., 2020 [78]
SWE (Temperature: 100–200 °C, Pressure: 25 × 10^5^ Pa)	GP (red and white varieties)	Gallic acid, Procyanidins, Malvidin-3-O-glucoside	TPC: 11.67 ± 1.67–72.52 ± 2.43 mg/g DW	Yammine et al., 2020 [135]
NaDES and PHWE	Tempranillo GP	Anthocyanins	Total Anthocyanin Content: 19.62–214.57 mg malvidin-3-O-glucoside eq./L	Loarce et al., 2021 [56]
UAE (Temperature: 80 °C, Time: 30 min) and β-CD	Muscat GP	Gallic acid, Catechin, Quercetin	TPC: 57.47 mg GAE/g DW	Alibante et al., 2021 [128]
SLE (Ethanol–Water (40/60); 16 h; In Dark)	Negramaro and Fiano whole GP and GP skins	Gallic acid, Caffeic acid, Catechin, Quercetin-3-O-glucoside, Anthocyanins	TPC: 36.8 ± 5.03–127.87 ± 8.03 mg GAE/g DW	Gerardi et al., 2021 [46]
MHG (Power: 400 W, Temperature: Approx. 80 °C, Time: Less Than 10 min)	Merlot and Cabernet Sauvignon GP	Gallic acid, Syringic acid, Quercetin	TPC: 118.1 mg GAE/g DW	Moro et al., 2021 [100]
NaDES and MAE	White GP	Gallic acid, p-Coumaric acid, Ferulic acid, Quercetin	TPC: 5.94 ± 0.29–43.73 ± 2.19 mg GAE/g DW	Cañadas et al., 2023 [125]
SLE (Ethanol–Water (1:1), Sonication: 20 min, Stirring: 2 h) with SPE	White and red GP	Catechin, Gallic acid, t-Caftaric acid, Quercetin, Kaempferol	TPC: 144–298 mg GAE/g DW (skins) and 327–540 mg GAE/g DW (seeds)	Guaita et al., 2023 [47]
NaDES and ASE (10 MPa, 90 °C)	Carménère red GP	Gallic acid, Caffeic acid, Quercetin	TPC: 33.39 ± 0.59–62.44 ± 1.67 mg GAE/g DW	Huamán-Castilla et al., 2023 [50]
SLE (Ethanol, HCl)	Touriga Nacional and Sousão GP	3-O-Caffeoylquinic acid, Quercetin-3-O-glucoside, Malvidin-3-O-glucoside	TPC: 44.93 mg GAE/g	Moutinho et al., 2023 [7]
UAE (Temperature: 20 °C, Time: 30 min) and MHG (Temperature: 80 °C, Time: 10 min, Power Density: 2 W/g) with SPE	Aglianico red GP	Catechin, Malvidin-3-O-glucoside chloride, Cyanidin chloride	TPC: 6.5 to 8.5 times higher in MHG	Crescente et al., 2023 [101]

## Abbreviations

ABTS: 22′-Azinobis(3-Ethylbenzothiazoline-6-Sulfonic Acid), ASE: Accelerated Solvent Extraction, β-CD: β-Cyclodextrin, CAT: Catalase, CE: Capillary Electrophoresis, COX-2: Cyclooxygenase-2, DAD: Diode Array Detector, DPPH: 22-Diphenyl-1-Picrylhydrazyl, DW: Dry Weight, FRAP: Ferric-Reducing Antioxidant Power, GAE: Gallic Acid Equivalent, GC: Gas Chromatography, GP: Grape Pomace, GPx: Glutathione Peroxidase, HBA: Hydrogen Bond Acceptor, HBD: Hydrogen Bond Donor, HPLC: High-Performance Liquid Chromatography, IFNγ: Interferon Gamma, IL-1β: Interleukin-1 Beta, IL-6: Interleukin-6, IL-8: Interleukin-8, IL-10: Interleukin-10, IL-18: Interleukin-18, iNOS: Inducible Nitric Oxide Synthase, LC: Liquid Chromatography, LPS: Lipopolysaccharide, MAE: Microwave-Assisted Extraction, MALDI-ToF: Matrix-Assisted Laser Desorption/Ionization–Time of Flight, MDA: Malondialdehyde, MHG: Microwave Hydrodiffusion and Gravity, MPO: Myeloperoxidase, MS: Mass Spectrometry, NaDES: Natural Deep Eutectic Solvents, NF-κB: Nuclear Factor Kappa B, NO: Nitric Oxide, PGE2: Prostaglandin E2, PHWE: Pressurized Hot Water Extraction, RNS: Reactive Nitrogen Species, ROS: Reactive Oxygen Species, RSS: Reactive Sulfur Species, RSM: Response Surface Methodology, SCFAs: Short-Chain Fatty Acids, SLE: Solid–Liquid Extraction, SOD: Superoxide Dismutase, SPE: Solid-Phase Extraction, SWE: Subcritical Water Extraction, TNF-α: Tumor Necrosis Factor Alpha, TPC: Total Phenolic Content, UV: Ultraviolet.
